# Hepatic Dysfunction in Hospitalized Patients with Acute Thyrotoxicosis: A Decade of Experience

**DOI:** 10.5402/2012/325092

**Published:** 2012-11-29

**Authors:** Richard M. Elias, Diana S. Dean, Gregory W. Barsness

**Affiliations:** ^1^Department of Internal Medicine, Mayo Clinic, 200 First Street SW, Rochester, MN 55905, USA; ^2^Division of Endocrinology, Mayo Clinic, 200 First Street SW, Rochester, MN 55905, USA; ^3^Division of Cardiovascular Diseases, Mayo Clinic, 200 First Street SW, Rochester, MN 55905, USA

## Abstract

Thyroid disease is a common condition, and thyroid hormone excess or deficiency is known to have wide-ranging effects on a variety of organ systems. Our objective is to describe the magnitude, biochemical features, and clinical characteristics of hepatic abnormalities in patients with acute thyrotoxicosis. We performed a retrospective review of all patients admitted to our institution between January 1, 1998 and December 31, 2008 with a discharge diagnosis of acute thyrotoxicosis excluding iatrogenic causes. The records of these patients were reviewed and data extracted regarding demographic, biochemical, and clinical data particularly relevant to liver function. Fourteen patients were identified of which eleven had liver studies performed. The majority (90.9%) had Graves disease. Nine of eleven patients (81.8%) had some degree of hepatic abnormality. Seven patients (63.6%) had an elevation in one or both transaminases, and two (18.2%) had isolated synthetic dysfunction as manifested as an elevated INR and/or decreased albumin without transaminitis. The mean magnitude of deviation from the normal range was greater in the transaminases as compared to bilirubin, INR, or albumin. Definitive treatment was radioiodine ablation in six cases (54.5%) and surgical thyroidectomy in two cases (18.2%). Noniatrogenic acute thyrotoxicosis requiring hospitalization is a rare condition which is most frequently caused by Graves disease. The majority of patients have disordered liver tests of a highly variable nature, making the recognition of this association important in the care of patients presenting with acute thyrotoxicosis.

## 1. Introduction

Thyroid disease is a common condition, and thyroid hormone excess or deficiency is known to have wide-ranging effects on a variety of organ systems. Liver failure in hyperthyroidism was first reported by Habershon in 1874 [1]. Hyperthyroidism has been known for the past seventy years to be associated with abnormalities of serum liver chemistry and histology [2, 3]. While mild derangements of hepatic enzymes have been recognized to occur frequently in states of both thyroid hormone excess and deficiency, the sometimes profound liver dysfunction noted in acute thyrotoxicosis is infrequently described in the literature and the etiology is not well understood [4]. Perhaps owing to the relative rarity of true thyroid crisis, defined as an acute, life-threatening exacerbation of hyperthyroidism with decompensation of one or more organ systems, discussion of this process and the potentially profound hepatic effects is limited to case reports [5–7]. The paucity of systematic assessment of the type and magnitude of liver dysfunction in this patient group is further complicated by the heterogeneity of the population, with a majority of cases arising secondary to a precipitant such as medications, most commonly amiodarone, or following thyroid surgery. We performed a retrospective review of adult patients admitted to Mayo Clinic Rochester over the past decade with noniatrogenic acute thyrotoxicosis in order to specifically investigate the nature and extent of hepatic abnormalities in these patients.

## 2. Methods

This research was approved by the Mayo Clinic Institutional Review Board (IRB ID no. 08-008253). A review was undertaken of all electronic discharge summaries for patients 18 years of age or older at our institution between January 1, 1998 and December 31, 2008. A search of discharge diagnoses including the terms “acute thyrotoxicosis,” “thyroid storm,” or “thyroid crisis” yielded 130 discharge documents. Fifty-two of these documents were found to be either duplicates or documentation of transfer from one hospital service to another during the same admission. The electronic medical records of the remaining patients were manually reviewed. In an effort to identify patients with clinically important and well-documented thyrotoxicosis as a cause of admission, additional patient records were excluded as follows: thirty patients were excluded because review indicated that hyperthyroidism was not a principal element of their admission, and they did not have clinical instability or organ dysfunction potentially attributable to acute thyrotoxicosis; eight admissions were excluded because they were for elective thyroidectomy in patients who had previously had acute hyperthyroidism; twenty-six patients were excluded because the principal etiology of their hyperthyroidism was determined to be amiodarone effect, making interpretation of liver function test (LFT) abnormalities difficult due to the potential for amiodarone to cause liver enzyme changes directly. This resulted in a pool of fourteen patients, which comprised the group for the review. Data was extracted from patient records including serum thyroid indices and LFTs as well as markers of hepatic synthetic function, namely, serum albumin and international normalized ratio (INR). The presence or absence of ascites was noted. In order to determine the extent to which liver abnormalities were secondary to high-output cardiac failure, echocardiogram and troponin data were recorded when available. As propylthiouracil (PTU) has been associated with cases of liver damage and failure, determination was also made as to whether the patient had been commenced on PTU prior to the liver testing. If patients were treated with PTU following testing, subsequent liver tests were assessed to determine if the values worsened after commencement of PTU.

## 3. Results

**Figure 1:**
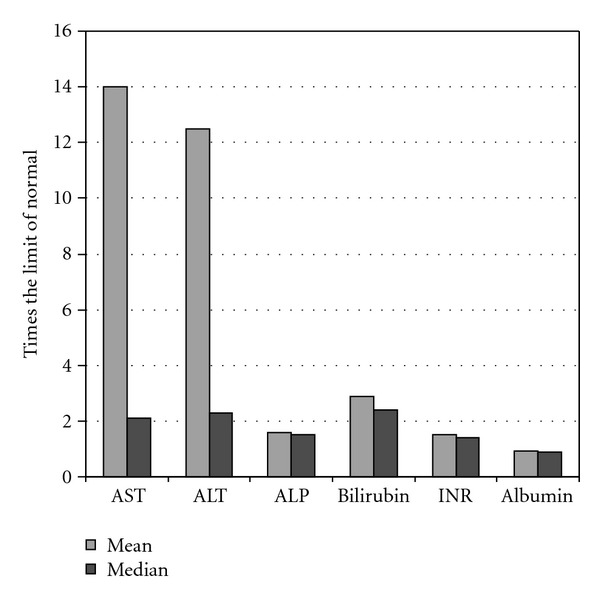
Mean and median degree of variation from the normal range of laboratory indices expressed as a multiple of the upper limit of normal (lower limit of normal for albumin). AST = aspartate aminotransferase; ALT = alanine aminotransferase; ALP = alkaline phosphatase; INR = International Normalized Ratio.

Fourteen patients were identified as having been admitted for de novo acute thyrotoxicosis over this period. Of this number, eleven patients (78.6%) had LFTs performed during their admission (Table 1). The average age at admission was 45 years (range 21–81 years). There was a slight female preponderance (54.5%). The etiology was thought to be Graves disease in 10 cases (90.9%) and unclear at the time of discharge in one. Two patients (18.2%) had known hyperthyroidism prior to this admission and had been nonadherent to their medication regimen. Ten patients had their INR checked and seven had an albumin level. Nine of eleven patients (81.8%) had some degree of hepatic abnormality as defined by elevated liver enzymes or INR, decreased albumin or evidence of ascites. Seven patients (63.6%) had an elevation in one or both of aspartate aminotransferase and alanine aminotransferase (AST and ALT), and two (18.2%) had isolated synthetic dysfunction as manifested as an elevated INR and/or decreased albumin without transaminitis. Six (54.5%) had elevated LFTs and impaired synthetic function whilst two (18.2%) were positive for LFTs, synthetic dysfunction, and ascites. Seven of the eight patients in whom alkaline phosphatase (ALP) was measured demonstrated an elevation. The mean and median laboratory values of patients with abnormal results and all of the patients respectively are listed in Table 2. The degree of variation from the upper limit of the normal range is represented in Figure 1. There did not appear to be a correlation between thyroid indices (thyroxine, triiodothyronine, TPO, or TRAB) and the degree of hepatic derangements. Abnormalities were also assessed on the basis of the degree of deviation from the normal range and the results are presented in Figure 1. Two patients (patients no. 5 and no. 10) had been taking PTU prior to measurement of LFTs. Patient no. 5 had been taking it for several months prior to admission and patient no. 10 for only several hours prior to LFT measurement. In both cases, PTU was continued and LFTs improved. Seven patients had a left ventricular ejection fraction (LVEF) measured by transthoracic echocardiography. LVEF was depressed (<55%) in five cases (71.4%). There did not appear to be a consistent relationship between magnitude of hepatic abnormalities although the patient (4) with the most profoundly abnormal liver tests was also the patient with the most depressed LVEF (20%). Definitive treatment ultimately consisted of surgical thyroidectomy in two cases (18.2%) and radioactive iodine in six (54.5%). One patient died during the index admission, and two others were lost to followup regarding their definitive management. Of the nine patients with hepatic abnormalities at baseline, follow-up liver tests were available in seven cases (patient no. 2 died during their index admission and patient no. 7 was lost to followup). The time period of follow-up ranged from 6 days to 27 months. In 6 of the 7 cases (77.8%) liver tests normalized. The mean and median time period between the index laboratory tests and the test demonstrating normalization were 8.5 months and 3 months, respectively. For only one patient (11) could liver tests not be shown to have normalized and in this case the available followup was only 6 days.

## 4. Discussion

It is known that thyroid hormones affect a variety of tissues via numerous established mechanisms. This investigation highlights the clinically important association between excess thyroid hormone and liver abnormalities. Documented acute thyroid crisis appears to be a rare condition, with only fourteen noniatrogenic cases identified at a large tertiary referral center over an eleven-year period. Of note, the majority of admissions labeled as acute thyrotoxicosis appear to be secondary to amiodarone with the majority of the remaining cases secondary to Graves disease. Importantly, most of these patients present with some degree of hepatic dysfunction and LFT abnormality which is highly variable in character and severity. This is in keeping with previous reports demonstrating that mild derangements in LFTs are common even in patients with subclinical hyperthyroidism but that hepatic failure is rare [4]. 

Examination of the LFT patterns in these patients demonstrates a high degree of variability. The most common LFT pattern is characterized by a transaminitis out of proportion to synthetic dysfunction. This is not invariably the case, however, with two patients demonstrating rises in INR without any marked abnormality of the transaminases and one patient with a mixed, predominately conjugated, hyperbilirubinemia and an elevated INR without transaminitis. Other investigators have noted a pattern of cholestatic liver dysfunction, with case reports documenting severe jaundice as the predominant clinical feature [6]. Others have noted rates in excess of 50% of jaundice and conjugated hyperbilirubinemia in uncomplicated hyperthyroidism in the presence of congestive heart failure, perhaps suggesting that circulatory congestion or relative perfusion insufficiency is more likely to be the etiology of hepatic abnormalities in less acutely ill patients, whereas direct thyroid hormone-mediated hepatocyte damage predominates in patients with acute thyrotoxicosis [8].

Notably, while all eleven patients comprising this study population had very depressed or undetectable thyroid-stimulating hormone levels, the circulating levels of thyroxine, free triiodothyronine, and antibodies to thyroid peroxidase varied considerably. There does not appear to be a correlation between the degree of thyroid function abnormalities and either the nature or magnitude of liver dysfunction, an observation that is in keeping with the results of previous investigations [8]. This lack of a “dose-dependent” relationship between thyroxine or triiodothyronine levels and liver dysfunction suggests that the notion of direct hypermetabolic pathways as the principal etiologic factor leading to liver damage is likely an oversimplification, with transporter-dependent and intranuclear transcriptional factors likely playing an important role. Given that the magnitude of hepatic abnormalities in our cohort of inpatients, who were admitted principally for acute thyrotoxicosis, is higher than that found in generally less acutely ill outpatients, it does seem that sicker patients with higher acuity hyperthyroidism have greater hepatic derangements. Specific risk factors for more profound liver disease in hyperthyroid patients remain incompletely understood.

The mechanism for an association between thyroid hormone excess and hepatic dysfunction is unclear and may be due either to indirect pathways or, alternatively, to direct hormone effects on the target organ, such as an interaction with nuclear receptors to modify gene expression or through receptors at the plasma membrane, mitochondria, and other extranuclear sites [9]. While these thyroid hormone effects on target cells remain incompletely elucidated, it is believed that some effects on hepatocytes are mediated by thyroid hormone receptors within the hepatocyte nucleus which alter the transcriptional activity of thyroid hormone-responsive genes [10]. As lipophilic substances, thyroid hormones readily enter cells, including hepatocytes, via diffusion, as well as via specific transporters present on both the plasma and nuclear membranes. Thyroid hormone increases cellular metabolism and ATP consumption via a mitochondrial mechanism, mediating changes in the permeability of the mitochondrial membrane and the rate of oxidative phosphorylation [11, 12]. Other hepatocyte systems known to be affected by thyroid hormones include the calcium regulation mechanism as well as the microsomal P450 system [13]. 

Investigations of the syndrome of resistance to thyroid hormone (RTH) have been indirectly instructive regarding the implications of thyroid hormone excess in the liver [14, 15]. Initially thought to be a deficiency of end-organ sensitivity to thyroid hormone, RTH is increasingly recognized as a spectrum of abnormalities related to receptor gene mutations, altered transport, and impaired metabolism of thyroid hormone. Impaired intracellular transport of thyroid hormone via the monocarboxylate transporter (Mct8), in particular, has been demonstrated to cause tissue-specific variation in triiodothyronine (T3) access characterized by impaired transport into cerebral cells but preserved transport into hepatocytes. When supranormal doses of triiodothyronine (T3) are administered to Mct8 knockout mice, markers of increased hepatocyte T3 included upregulation of thyroid hormone responsive genes and increased serum alkaline phosphatase [16]. While generally demonstrating the potential direct effects of thyroid hormone excess on hepatocytes, RTH does not appear to have been an important feature in our patient group, given that they all had markedly suppressed thyroid stimulating hormone.

Apart from direct hepatocellular effects, thyrotoxicosis may cause secondary hemodynamic insults that can perturb liver function. Congestive heart failure exacerbation, often secondary to atrial fibrillation but also described in sinus tachycardia, has a recognized association with hyperthyroidism. Studies of this association have found it to be an uncommon manifestation of hyperthyroidism generally, although research specifically investigating patients with acute thyrotoxicosis is limited [17]. It seems likely that congestive heart failure is more common in this population and serves as a potential confounding variable as it is a well-recognized cause of acute liver dysfunction and disordered LFTs. In our patient group, there does not appear to be a correlation between left ventricular ejection fraction (LVEF) and degree of hepatic dysfunction. Of the three patients with LVEF less than 30%, only one demonstrated a marked transaminitis. Interestingly, one patient with a diminished LVEF demonstrated essentially normal transaminases but an elevated INR. Conversely, of the three patients with the most profound hepatic abnormalities, only one had impairment of LVEF. This lack of correlation between left ventricular function and degree of hepatic abnormalities differs from previous research which found that hepatic abnormalities were greater in patients with CHF. However, that study investigated a more well-compensated cohort of patients with hyperthyroidism with markedly less disordered LFTs. This suggests that the etiology of the deranged LFTs seen in patients with milder or subacute hyperthyroidism is more dependent on the longer term cardiac effects of thyroid hormone excess [8]. 

It has been recognized since studies performed in the 1950s that splanchnic metabolism, as measured by oxygen consumption, is elevated in hyperthyroidism [18]. This increased oxygen extraction does not appear to be accompanied by a concomitant increase in splanchnic blood flow, with increased cardiac output preferentially directed toward skin, skeletal muscle, and possibly the renal vasculature. In a study performed by Myers and colleagues, arteriovenous oxygen gradient was found to be increased to 50% above normal in hyperthyroidism. This finding, together with pathologic findings of centrilobular necrosis found in older autopsy studies, suggests that in cases of more prolonged untreated hyperthyroidism, relative hepatic anoxia is a principal mechanism of injury and circulatory collapse the mechanism of death [3, 19]. More recent studies of hepatic pathology in hyperthyroidism based on biopsy specimens in living subjects suggested subtler changes of cellular architecture, most prominently cytoplasmic clarification, decrease in ribosome numbers and density of the mitochondria and various nuclear changes such as hyperchromatism, karyolysis and alterations of nuclear size, supporting the notion of complex influences of thyroid hormones on a variety of hepatocyte functions, and modulation of a variety of target receptors [8, 20, 21].

While this analysis represents the largest recent report detailing liver dysfunction in the setting of thyrotoxicosis, there are several limitations to this study. Broad applicability of these findings is limited by the disparate definitions of acute thyrotoxicosis, thyroid storm, or thyroid crisis in the literature, including definitions requiring that the episode be acutely life-threatening, and others dependent on decompensation of one or more organ systems to meet the definition [22, 23]. Although there exists a set of criteria for “thyroid storm” published by Burch and Wartofsky, based on a physiological scoring system, as a guide to diagnosis, ultimately there are no consensus criteria and the diagnosis is made largely on clinical grounds [24]. The patient group in this study was based on a retrospective review of the discharge diagnoses of the treating physician rather than objective criteria for a diagnosis of acute thyrotoxicosis, and this may partly explain the heterogeneity of the presentations, which ranged from short, lower acuity admissions to severely ill patients with multiorgan failure requiring intensive care. Rather than a methodologic flaw, however, it is reasonable to draw from this contemporary observation the notion that thyrotoxicosis is a disorder with a highly variable presentation and one for which we remain poorly equipped to anticipate the clinical course and outcomes on the basis of objective clinical and biochemical parameters. 

Another potential confounder is the increasing recognition of the association between autoimmune thyroid disease and autoimmune liver disease. While none of the patients in our study had diagnoses of autoimmune hepatitis, primary sclerosing cholangitis or primary biliary cirrhosis, testing was not universally conducted to specifically exclude this possibility. Similarly, none of the included patients had known viral or other primary liver diseases but these potential confounders cannot be excluded. 

## 5. Conclusions

While hyperthyroidism is a common condition, acute, severe thyrotoxicosis without an iatrogenic precipitant is uncommon and most frequently associated with Graves disease. These patients are present with liver abnormalities which are variable in character and magnitude but appear to have a hepatitic predominance. Evidence of thyroid dysfunction should be sought in cases of unexplained hepatic abnormalities, particularly transaminitis. There remains lacking a standardized mechanism for diagnosis and grading of acute thyrotoxicosis and the development of this would facilitate further systematic study of the end-organ effects of this condition. 

## Figures and Tables

**Table 1:** Demographic, laboratory, echocardiographic results, and propylthiouracil use by patient.

Patient number	1	2	3	4	5	6	7	8	9	10	11
Age	81	50	55	21	26	23	42	42	56	55	50
Gender	F	F	F	M	F	M	M	M	M	F	F
TSH (mIU/L) [NR 0.3–5.0 mIU/L]	0.02	<0.01	<0.01	<0.01	<0.01	<0.01	<0.01	<0.01	0.01	<0.01	0.01
Thyroxine (total; mcg/dL) [NR 5–12.5 mcg/dL]	NP	NP	11.5	NP	23.2	22.3	NP	NP	16.2	NP	NP
Thyroxine (free; ng/dL) [NR 0.8–1.8 ng/dL]	10	4.6	6	7.7	7	>12	5.4	9.4	3.2	5	3.6
Triiodothyronine (ng/dL) [NR 80–190 ng/dL]	NP	NP	462	385	NP	359	NP	NP	150	NP	NP
Free T3 (pg/mL) [NR 2.0–3.5 pg/mL]	20	8.6	NP	17.6	10.3	>20	17.6	NP	NP	12.3	43 (reverse)
TPO (IU/mL) [NR < 9.0 IU/mL]	<20	287	NP	>950	NP	4420	NP	3017	706	NP	NP
TRAB (%) [NR < 16%]	82	31	66	NP	NP	93	46	75	52	NP	53
TSI index (%) [NR ≤ 1.3%]	NP	NP	NP	7.3	NP	NP	NP	NP	NP	5	2.6
											
AST (U/L) [NR 8–48 U/L]	76	978	636	2850	39	30	82	34	99	52	69
ALT (U/L) [NR 7–55 U/L]	66	920	841	1895	41	27	102	53	70	41	44
ALP (U/L) [NR 45–115 U/L]	135	252	136	180	129	NP	97	NP	194	NP	162
Bilirubin (Total/Dir.; mg/dL) [NR 0.1–1.0/0.0–0.3 mg/dL]	NP	1.2/0.6	0.9/0.7	5.6/1.8	0.4/0.1	NP	0.7/0.2	NP	1.2/0.7	NP	3.6/2.5
INR [NR 0.8–1.2]	NP	1.2	1.4	2.4	1.1	1.0	1.0	1.0	1.1	1.7	1.7
Albumin (g/dL) [NR 3.5–5.0 g/dL]	NP	2.9	3.6	3.6	NP	NP	3.3	NP	3.4	3.1	3.0
Ascites	N	N	N	Y	N	N	N	N	Y	N	N
LVEF by ECHO	NP	48%	65%	20%	NP	NP	68%	45–50%	22%	29%	NP
Troponin T (ng/mL)	NP	12.38	0.07	<0.01	<0.01	<0.01	<0.01	0.11	<0.01	NP	NP
											
PTU prior to LFTs?	N	N	N	N	Y	N	N	N	N	Y	N
PTU after LFTs?	Y	Y	N	N	Y	Y	N	N	Y	Y	Y
LFTs worsened?	N	N	N/A	N/A	N	N	N/A	N/A	N	N	N

Y: yes, N: no, NP: not performed, N/A: not applicable.

**Table 2:** Mean and median values of hepatic indices.

		AST (U/L)	ALT (U/L)	AST : ALT	ALP^1^ (U/L)	Bilirubin (total/dir.; mg/dL)	INR	Albumin (g/dL)
All patients (*n* = 11)	Mean	450	373	1.11	161	1.9/0.9	1.4	3.3
	SD	854.04	603.74	0.31	48.04	1.93/0.88	0.45	0.28
	Median	76	66	1.11	149	1.2/0.7	1.15	3.3

Patients with abnormalities	Mean	605	564	1.18	170	2.9/1.3	1.8	3.0
	SD	969.73	700.19	0.34	43.83	2.13/0.85	0.42	0.21
	Median	90.5	102	1.15	162	2.4/0.7	1.7	3.1

^1^None of the patients had isoenzyme analysis to differentiate hepatic from bone ALP.
